# Peripheral Nerve Activation Evokes Machine-Learnable Signals in the Dorsal Column Nuclei

**DOI:** 10.3389/fnsys.2019.00011

**Published:** 2019-03-20

**Authors:** Alastair J. Loutit, Mohit N. Shivdasani, Ted Maddess, Stephen J. Redmond, John W. Morley, Greg J. Stuart, Ingvars Birznieks, Richard M. Vickery, Jason R. Potas

**Affiliations:** ^1^School of Medical Sciences, UNSW Sydney, Sydney, NSW, Australia; ^2^Eccles Institute of Neuroscience, The John Curtin School of Medical Research, Australian National University, Canberra, ACT, Australia; ^3^Graduate School of Biomedical Engineering, UNSW Sydney, Sydney, NSW, Australia; ^4^Bionics Institute, East Melbourne, VIC, Australia; ^5^UCD School of Electrical and Electronic Engineering, University College Dublin, Dublin, Ireland; ^6^UCD Centre for Biomedical Engineering, University College Dublin, Dublin, Ireland; ^7^School of Medicine, Western Sydney University, Sydney, NSW, Australia

**Keywords:** machine learning, tactile, proprioception, somatosensory, lateralization, neural prosthesis, gracile nuclei

## Abstract

The brainstem dorsal column nuclei (DCN) are essential to inform the brain of tactile and proprioceptive events experienced by the body. However, little is known about how ascending somatosensory information is represented in the DCN. Our objective was to investigate the usefulness of high-frequency (HF) and low-frequency (LF) DCN signal features (SFs) in predicting the nerve from which signals were evoked. We also aimed to explore the robustness of DCN SFs and map their relative information content across the brainstem surface. DCN surface potentials were recorded from urethane-anesthetized Wistar rats during sural and peroneal nerve electrical stimulation. Five salient SFs were extracted from each recording electrode of a seven-electrode array. We used a machine learning approach to quantify and rank information content contained within DCN surface-potential signals following peripheral nerve activation. Machine-learning of SF and electrode position combinations was quantified to determine a hierarchy of information importance for resolving the peripheral origin of nerve activation. A supervised back-propagation artificial neural network (ANN) could predict the nerve from which a response was evoked with up to 96.8 ± 0.8% accuracy. Guided by *feature-learnability*, we maintained high prediction accuracy after reducing ANN algorithm inputs from 35 (5 SFs from 7 electrodes) to 6 (4 SFs from one electrode and 2 SFs from a second electrode). When the number of input features were reduced, the best performing input combinations included HF and LF features. Feature-learnability also revealed that signals recorded from the same midline electrode can be accurately classified when evoked from bilateral nerve pairs, suggesting DCN surface activity asymmetry. Here we demonstrate a novel method for mapping the information content of signal patterns across the DCN surface and show that DCN SFs are robust across a population. Finally, we also show that the DCN is functionally asymmetrically organized, which challenges our current understanding of somatotopic symmetry across the midline at sub-cortical levels.

## Introduction

The brainstem DCN are one of the first processing centers for ascending somatosensory information, preceding conscious perception in the somatosensory cortex. There has been a recent resurgence of interest in the DCN, to investigate how somatosensory signals are transformed as they ascend the neuraxis to the neocortex (Witham and Baker, 2011; Bengtsson et al., 2013; Jörntell et al., 2014), and to investigate the DCN as a potential neuroprosthetic target to restore somatosensation following spinal cord injury (Richardson et al., 2015, 2016; Sritharan et al., 2016; Suresh et al., 2017). However, there is still little known about how afferent signals are processed in this region.

We previously characterized electrical signals acquired from the DCN surface in response to peripheral nerve stimulation demonstrating a range of highly characteristic SFs (Loutit et al., 2017). These SFs are significantly different when evoked from peripheral nerves with compositions of afferents that innervate different peripheral structures – either primarily cutaneous (sural nerve), or a mixture of cutaneous and deep structures (peroneal nerve) – and can, therefore, reveal information about the type of sensory input arising from the periphery. The reproducibility and conservation of these SFs within and across different animals suggests that they might be indicative of physiologically relevant neural processes (Loutit et al., 2017).

Features of particular interest include two that represent HF events and three comprising LF events. LF components of electrocorticographic (ECoG) potentials, similar to those investigated here, have been attributed to arise from slow network activity related to synaptic events (Buzsáki et al., 2012), while the HF components are believed to represent multiunit spiking activity, generated from axonal action potentials (Logothetis, 2003). While most studies have focused on single-cell spiking dynamics of DCN cells (Canedo et al., 1998; Nuñez et al., 2000; Rowe, 2002; Bengtsson et al., 2013; Hayward et al., 2014; Jörntell et al., 2014), there is little information on multi-unit activity or peripherally evoked LF events in the DCN.

In the present study, we aimed to determine if DCN surface potential SFs contain information that can accurately identify the site where a peripheral sensory signal originated. To achieve this, we quantify how learnable classes of input features are, as determined by a ML algorithm’s success in making accurate classifications (Valiant, 1984; Schapire, 1990), which we define here as *feature-learnability*. Feature-learnability is distinct from Vapnik–Chervonenkis (VC) theory (Vapnik and Chervonenkis, 1971) or probably approximately correct (PAC) theory (Valiant, 1984) that aim to determine whether the problem faced by an ML algorithm is machine-learnable (statistically learnable) (Blumer et al., 1989), and does not concern whether its machine-learnability is decidable (Ben-David et al., 2019). Rather, we use feature-learnability to quantify and rank the electrode positions and SFs with the most relevant information for classifying the source of sensory input. Feature-learnability permits us to focus on the information content of the signals, as opposed to the performance of the ML algorithm.

Here, we record electrically induced peripheral signals from the DCN with a surface multielectrode array (sMEA) and extract features from established LF and HF signal frequency bands. We train supervised back-propagation ANNs to classify the nerve evoking a DCN response based on combinations of SFs derived from selected electrodes. We confirm the reproducibility and robustness of DCN nerve-evoked SFs, both within and across different animals, and show that a combination of LF and HF features are required for maximizing classification accuracy with a minimized set of electrodes and SFs. We also confirm that feature-learnability reliably reflects the statistical differences in the physiological characteristics of DCN signals, and demonstrate that high ML accuracy relates to asymmetrically arranged SFs across the surface of the DCN (Loutit et al., 2017).

## Materials and Methods

### Animals

All procedures were approved by the Australian National University Animal Experimentation Ethics Committee (A2014/52) and adhered to the Australian code of practice for the care and use of animals for scientific purposes. Seven (*n* = 7) 8-week-old male Wistar rats (325–420 g; Australian Phenomics Facility, Canberra, ACT, Australia) were used in this study. Animals were housed individually, or with up to 3 rats per cage, on a 12-h light-dark cycle, with access to food and water *ad libitum*.

### Surgery

**FIGURE 1:**
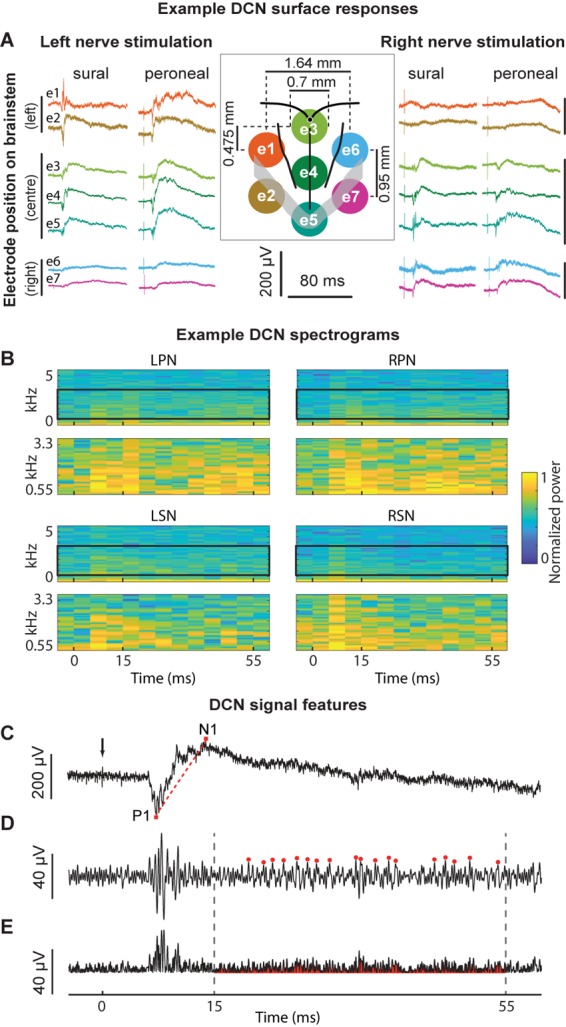
Example signals, signal processing, and feature extraction of nerve-evoked potentials recorded from the DCN. **(A)** An example set of recordings from a single animal. Stimuli were presented to the left or right sural or peroneal nerves and evoked potentials recorded on the surface of the DCN. The central insert illustrates the sMEA electrode configuration (e1–e7) and size relative to anatomical landmarks (obex indicated by the small black filled circle; midline, gracile/cuneate nuclei borders indicated by black boundaries; major vessels indicated by gray overlay). The colors of the recorded signals match those of their electrode (also indicated in far-left traces). **(B)** Spectrograms of example signals evoked by each of the four nerves recorded at e4. Top traces in each quadrant show spectral power between 1 and 5000 Hz. Black boxes outline the frequency HF band of interest from 550 to 3300 Hz, which are expanded in the lower plots of each quadrant. Spectral power was normalized between 0 and 1 for each trace to facilitate comparison between the nerves. **(C)** An example of an individual sural nerve-evoked DCN surface potential raw signal (10 kHz low-pass filter; positive potentials shown as downward deflections). Arrow indicates stimulus artifact. P1 indicates the peak of the positive deflection and N1 indicates the peak of the negative deflection. The *P1N1 amplitude* SF was measured as N1 – P1 (μV). The gradient of the red dashed line demonstrates the *P1N1 slope* SF. The *N1 latency* SF was measured as the time from the stimulus artifact (black arrow) to the peak of the negative deflection (N1). Same signal as **(C)** after band-pass filtering (550–3300 Hz). Red dots indicate event detection used to quantify the *HF peak count* SF, defined as peaks > 3 standard deviations above background noise. **(E)** Signal in **(D)**, rectified. The red shaded region underneath the signal indicates the *HF integral* SF. Dashed lines indicate time window from 15 to 55 ms for extraction of the HF peak count **(D)** and HF integral **(E)** SFs. DCN, dorsal column nuclei; e1–e7, electrode 1 to electrode 7; sMEA, surface multi-electrode array; SF, signal feature.

Animals were anesthetized with urethane (1.4 g/kg i.p). The brainstem was exposed between the foramen magnum and the C1 vertebra and the dura and arachnoid mater excised. In most cases the C1 vertebra was partially cut away with rongeurs (World Precision Instruments) to create more space for placement of the sMEA. The left and right sural and peroneal nerves were isolated and prepared for electrical stimulation as previously described; see Loutit et al. (2017) for full details of the surgical procedures and electrophysiology setup of hindlimb nerves. Seven simultaneous recordings across the surface of the DCN were performed by adapting a sMEA (*Nucleus 22 Auditory Brainstem Implant*, Cochlear Ltd. (see Chelvanayagam et al., 2008 for details), which was symmetrically aligned over the brainstem (Figure 1A, central insert). A flexible plastic rod, attached to a micromanipulator, was used to lightly press the sMEA onto the brainstem to hold it in place and facilitate stable recordings.

### Stimulation, Recording, Electrode Nomenclature and Signal Processing

Electrical stimulation and recordings were driven and acquired, respectively, by the same data acquisition system (PowerLab 16/35, LabChart Pro software Version 8.1.1, ADInstruments, Bella Vista, NSW, Australia). Supramaximal single electrical monophasic pulses were used to stimulate sural and peroneal nerves on the right and left side of the body (RSN, LSN, RPN, and LPN) whilst responses were simultaneously recorded from the ipsilateral sciatic nerve and the DCN surface as previously described (Loutit et al., 2017). Twenty sets of stimuli (11 trials at 0.53 Hz per set) were delivered as current pulses with 0.05 ms pulse-widths and 0.7–1.0 mA amplitudes, which were determined to be 10–20% above maximal stimuli for all nerves. DCN signals were acquired from the seven electrodes on the sMEA (Figure 1A) and filtered (50 Hz notch filter; 10 kHz low-pass filter) through custom built amplifiers before being digitally recorded at a sample rate of 40 kHz. There was no ringing observed from filtering in the DCN signals which had onset latencies >5 ms. The seven electrodes were referred to from rostral to caudal as follows: left side electrodes: e1, e2; midline electrodes: e3, e4, e5; right side electrodes: e6, e7 (Figure 1A). Combinations of electrodes are referred to in sets, e.g., electrodes e1, e4 and e5 combined are referred to as e{1, 4, 5} and all seven electrodes are referred to as e{1–7}.

### Signal Feature Extraction

Previously, we inspected time-frequency plots and observed nerve-dependent differences in the HF band between 550 and 3300 Hz of DCN surface signals (Loutit et al., 2017). Here, time-frequency plots were generated using the *spectrogram* function (MATLAB), with 10 ms rolling windows (5 ms overlap). Spectrograms were generated between 1 Hz and 10 kHz, to show the power spectral density of the raw signals (plotted from 1 Hz to 5 kHz), and between 550 and 3300 Hz to show the HF components of DCN surface responses (Figure 1B). These plots confirmed that the sural and peroneal nerves contain different information in the 550–3300 Hz HF band.

The extraction of each feature is described in Figure 1C–E. We selected three SFs related to the LF DCN waveform extracted directly from individual raw signals (Figure 1C): the latency to the peak of N1 (N1 latency), the slope of a line from the peak of P1 to the peak of N1 (P1N1 slope), and the amplitude from the peak of P1 to the peak of N1 (P1N1 amplitude). Two features were extracted from the HF filtered component of individual signals: the total number of HF events between 15 and 55 ms post-stimulus (HF peak count; Figure 1D), and the total integral of the rectified HF signal between 15 and 55 ms post-stimulus, less background noise (HF integral; Figure 1E). HF events were defined as peaks >3 standard deviations above the background noise (Figure 1D). Background noise was calculated from a 10 ms window (−15 ms to −5 ms pre-stimulus). Signal processing and analyses were performed offline (MATLAB version R2016b, MathWorks). Stimulation artifacts were reduced to background noise-levels to avoid ringing before signals were filtered to extract HF components (bandpass 550–3300 Hz; 10-order Butterworth filter, using a zero-phase response filter: *filtfilt* function, MATLAB; Figure 1D).

To test if any of the five input features represented similar DCN signal information, we performed Pearson’s product-moment correlations for each pair of features, extracted from all signals recorded across animals.

### Machine Learning and Feature-Learnability

The set of five SFs, extracted from each of the seven sMEA electrodes, was paired to the respective nerve being stimulated, thus generating input/output pairs for subsequent ML. We used a supervised learning classification algorithm from the MATLAB Neural Network Toolbox (version 2016b, Mathworks). This ANN was constructed with the *patternnet* function using a gradient descent with momentum and adaptive learning rate backpropagation training function (*traingdx*). The hidden layer activation function was a hyperbolic tangent sigmoid transfer function (*tansig*), and a softmax transfer function (*softmax*) was used in the output layer. These functions are commonly used for classification of this nature as they are adept at quantifying relevant information content hidden within a complex dataset, by resolving convoluted variable interactions and robustly mapping them to target classes (Szegedy et al., 2014; Carlini and Wagner, 2017). However, other ML approaches are equally valid for this purpose. Analyses were split into three sets: (i) using all five SFs from all seven electrodes, (ii) using all five SFs extracted from subsets of the seven electrodes, and (iii) using subsets of SFs extracted from subsets of electrodes. Inputs and target data were normalized between −1 and 1 prior to dividing into training, validation and test subsets, as per the *patternnet* default settings. In all analyses, the hidden layer was fixed at 20 neurons and the output layer had 4 neurons corresponding to the stimulated nerve targets (RSN, LSN, RPN, and LPN). The task for the ANN was thus to reliably determine which of the 4 nerves had been stimulated using different inputs. We started with an ANN that had 35 neurons in the input layer (5 SFs for each of the 7 electrodes), while the number of input neurons varied for the other analyses. Cross-validation for a given analysis configuration (choice of SF and electrode input features) was performed as follows: ML was repeated 10 times with different seed states each time to alter the ANN initial conditions and allocation of data into training, validation and testing subsets. Of the 10-repeated training/validation/testing cycles, the mean of the test results was used to establish a confusion matrix that represented stable learning outcomes. The mean of correct classifications for all possible outputs (i.e., *n* = 4 possible nerves) was used to report the feature-learnability outcomes of the supervised learning for each approach. Feature-learnability therefore provides: (i) a value for the mean prediction accuracy of all trained ANN outputs, and (ii) a range, represented by the SEM, that provides a measure of the variability of accuracy across the four possible outcomes (i.e., RSN, LSN, RPN, and LPN).

#### Feature-Learnability, Benchmark and Ranking Within and Across Animals

For the first set of analyses, three different approaches were used to train, validate and test the neural networks. All three approaches calculated the feature-learnability from the mean result of 10 repeated training/testing cycles, under different random starting conditions. Three approaches to determine feature-learnability were employed. The first was the Within Individual Animal approach (WIA) in which, input/output pairs, from 800 to 880 stimuli, from all four nerves were generated for each individual animal (*n* = 7) and were randomly sequenced and assigned into training, validation and testing data sets in the proportions of 70, 15, and 15%, respectively. In the second PP approach, input/output pairs from all four nerves were pooled from all 7 animals (5600–6160 total stimuli) and were randomly sequenced and assigned into training, validation and testing data sets in the same proportions as above. In the third Leave-One-Out (LOO) approach, training and validation were performed on input/output pairs randomly sequenced and assigned into training (70%) and validation (30%) sets from 6 animals, and testing was performed on the remaining animal (100%). Each of the 7 animals were examined as the test data set once. The feature-learnability benchmark (gold standard) was derived from the WIA approach, while the PP and LOO approaches explored the extent to which the learned features generalized across animals, and the LOO approach explored feature similarity of individual animals, in contrast to the remaining animals. Subsequent experiments made comparisons with the learnability benchmark.

“Near-benchmark” is defined as learnability that is not significantly different to that of the benchmark. Ranking of feature-learnability was performed by ordering the feature-learnability with respect to the greatest mean followed by the smallest SEM, i.e., in cases where two ANN configurations resulted in identical mean feature-learnability, the configuration with the smaller SEM outranked the larger. Ranking was independent of significant differences between two feature-learnability comparisons.

#### Electrode and Signal Feature Contribution to Feature-Learnability

In the second set of analyses (electrode contribution), all SFs acquired from single, and combinations of two or more electrodes, were incorporated into the ANN to compare how these features, from different electrodes, contribute to feature-learnability. In these ANNs, depending on the number of electrodes included, there were 5, 10, 15, 20, 25, 30, or 35 neurons in the input layer (i.e., 5 input SFs per electrode), and the hidden (20 neurons) and output (4 neurons) layers were identical to the ANN used for the learnability benchmark configuration (i.e., the first approach of first set of analyses described above).

In the third set of analyses (SF contribution), we tested the prediction accuracy of each of the SFs individually, and pairs of SFs, when quantified from single electrodes, three electrodes and all seven electrodes. If only single SFs were tested, there were either 1, 3, or 7 neurons in the ANN input layer (1 feature extracted from 1, 3, or 7 electrodes, respectively); for pairs of features, there were 2, 6, or 14 neurons in the ANN input layer (2 features extracted from 1, 3, or 7 electrodes, respectively).

The WIA approach, which determined the feature-learnability benchmark, was applied to both the electrode contribution and SF contribution sets of analyses. Thus, ML was performed on each individual animal and confusion matrices were averaged to generate a single confusion matrix that represented the set of seven animals. Feature-learnability was then derived from the mean and SEM of the diagonal from this matrix.

#### Input Minimization Feature-Learnability

In the final set of ANN analyses, we sought to determine the minimum number of ANN inputs required to achieve feature-learnability that was not significantly different to the benchmark. We applied two strategies: (i) to minimize the total number of inputs, regardless of the number of electrodes; and (ii) to minimize the total number of inputs and the number of electrodes. First, we ranked all single SF/electrode combinations from highest to lowest feature-learnability: Each individual SF/electrode combination was presented as the single input to an ANN with fixed hidden (20 neuron) and output (4 neuron) layers, the feature-learnability determined, and ranked from highest to lowest. The first input minimization strategy defined the sequence of SF/electrode combinations (next-best inputs) in their feature-learnability rank order. The second input minimization strategy ranked the electrodes based on the mean feature-learnability of all SFs on each respective electrode, and next-best inputs were derived from the SF rank-order within a given electrode. Thus, the second input minimizing strategy prioritized SF inputs within electrodes over the overall SF/electrode rank order; i.e., all SFs of the highest-ranked electrode were sequenced before SFs of a lower-ranked electrode, even if their individual SF/electrode combination feature-learnability was greater.

An exhaustive selection method that tested all SF/electrode combinations was not feasible, as the possible feature combinations would exceed 2 million. We therefore applied an adapted sequential forward searching (SFS) algorithm, first used by Whitney (1971) and well described by Pudil et al. (1994), for both input minimization strategies. Our approach differed from the typical SFS algorithm in that discarded SF/electrode combinations from each round of testing were re-tested in the next round, after a new best feature subset was established. The SFS algorithm was terminated when near-benchmark feature-learnability was attained.

#### Feature-Learnability Mapping

Feature learnability mapping was performed using all 5 SFs. The mean and SEM feature-learnability produced from seven ANNs, each using SFs from one of the seven electrodes, were plotted as a surface in 3D space over 2 mm × 2 mm generic DCN maps for each animal. Feature-learnability surfaces (one surface for the mean classification performance and another for the SEM), that passed through each of the 7 data points, were interpolated between the 7 data points onto a 7 × 7 mesh grid using an optimized thin plate spline function (Wahba, 1990). The surfaces for each animal were z-plane stacked following adjustments to account for left-right and rostro-caudal shift of the sMEA in each animal, and a mean for each grid coordinate across the *z*-stack was calculated to produce feature-learnability maps representing the average for the mean surface and the SEM surface of all animals.

#### Feature-Learnability Error and Machine Learning Error

Feature-learnability error provides insight into non-significant loci as potential errors for ANN classification, and was defined as [100 – feature-learnability]. ML error was used for the analysis of SF side dominance and was defined as a measure of the ML algorithm prediction inaccuracy, calculated as [100 – ML accuracy] for any combination of SF/electrode inputs. The e4 mean ML error refers to the average of ML errors from a single SF/e4 combination between two nerves of a bilateral nerve pair, incorrectly classified as their counterpart nerve, e.g., the mean of RPNs classified as LPNs plus LPNs classified as RPNs. An above chance level of the e4 mean ML error therefore indicates that the error of both nerves averaged greater than 25%. The e4 mean ML error is expressed for individual animals and SFs, whereas the feature-learnability error is expressed as an average across all animals.

### Electrode Shift Measurements

To determine where the electrode array was placed in each experiment, we measured the distance of the two left electrodes (e1 and e2) and right electrodes (e6 and e7) from the midline, and the rostro-caudal distance of the rostral edge of e3 from obex. Images were taken of the brainstem surface using a microscope camera both before and after the electrode was positioned. These images were stacked over each other using an image analysis software (ImageJ, version 1.51k). Lines were drawn along the midline of the brainstem, perpendicular from the midline to the most medial edge of each of the four side electrodes (e1, e2, e6, and e7), and rostro-caudally from the rostral edge of e3 to obex. If the array was perfectly placed in line with the brainstem midline, then the measurement from e1, e2, e6, and e7 to the midline would be 470 μm. We subtracted the electrode shift measurements from 470 μm. If 470 μm minus e1- and e2-to-midline measurements were negative, this indicated that the electrode was shifted to the left of the midline, and vice versa for the right electrodes.

### Non-significant Loci

To demonstrate that classification errors generated by the ANN were consistent with statistical observations of the input SFs, we used a statistical approach to identify likely occurrences for potential ML classification errors. We modeled DCN responses from individual electrodes in response to stimulation from each of the four nerves (each stimulated 200–220 times). For each animal and SF, we then generated a linear model to determine if there were significant differences in SF magnitudes for each electrode. Failures to reach a significant difference (*p* > 0.05) in SF magnitudes between two nerves were deemed as *non-significant loci* and assigned to one of 5 possible categories to indicate the two nerve-evoked responses that were not significantly different: (i) LSN vs. RSN; (ii) LPN vs. RPN; (iii) RPN vs. RSN; (iv) LPN vs. LSN; (v) LPN vs. RSN or RPN vs. LSN.

### Statistical Analysis

All statistical analyses were carried out using R (R Core Team, 2016; version 3.4.4) through the RStudio integrated development environment (version 1.1.442). Paired *t*-tests, one-way, and two-way ANOVAs with Tukey *post hoc* adjustments were used where stated to compare ANN classification accuracy between two specific nerves (such as ipsilateral nerves or bilateral nerve pairs) or multiple comparisons, respectively. To compare ANN performance for individual electrodes, combinations of electrodes, and individual SFs, we used LMEMs (*lmer* function in R) from the *lmerTest* package (Kuznetzova et al., 2016). When significant main effects were found, *post hoc* least squares mean differences (*difflsmeans* function in R) were calculated with Satterthwaite’s approximation to degrees of freedom, and *p*-value adjustments for multiple comparisons were performed by a method controlling the FDR (*post hoc*; R: *p.adjust* function; *BY* option). To determine the most efficient electrode combinations, we compared each combination to the benchmark using paired *t*-tests. To obtain correlation coefficients of ANN input features (see section “Results,” Feature-learnability benchmark: five SFs across seven electrodes), and to determine non-significant loci of input features, LM were created using the R *lm* function (*stats* package). When main effects were found for LMs, least square means comparisons were made with Tukey *post hoc* adjustments. Side dominance comparisons at the single e4 electrode were made using LMs and Pearson’s Chi-squared tests, where appropriate, while Student’s *t*-tests were used for comparing the TRM side dominance. All LMs, and LMEMs were verified by inspection of residual and QQ plots. Where ceiling effects (left skew) were found in ANN output data (values represented as percentages between 0 and 100), *logit* transformations, from the *car* package (Fox et al., 2016), were applied to the data. All data are expressed as means ± SEM. Sample numbers (*n*-values) are indicated in figures. Probabilities of *p* < 0.05 were deemed significant.

## Results

### Feature-Learnability Benchmark: 5 Signal Features Across 7 Electrodes

A representative example of raw DCN signals recorded from 7 electrodes evoked by left and right sural and peroneal nerves, and a schematic (to scale) representation of the electrode array configuration are shown in Figure 1A. Raw signal amplitudes were generally larger on midline electrodes and/or those ipsilateral to the nerve undergoing stimulation.

To establish a feature-learnability benchmark, we maximized the classification accuracy of an ANN using SFs from individual animals. Four SFs (N1 latency, P1N1 slope, HF peak count and HF integral, examples shown in Figure 1C–E) were previously shown to be significantly different between sural and peroneal nerve-evoked responses (Loutit et al., 2017), and were therefore chosen for the present study to facilitate correct predictions of the nerve type (sural or peroneal). Two SFs (P1N1 amplitude and HF integral) have previously been shown to demonstrate ipsilateral dominance with respect to response magnitudes (Loutit et al., 2017) and were therefore chosen to facilitate predictions of the correct side of the body. Pearson’s correlations between all pairwise combinations of SFs for all electrodes and all nerves were found to be highest between HF integral and HF peak count (Table 1), suggesting that these SFs are likely to contribute redundant information to the ANN. However, both these SFs were included in feature-learnability experiments to establish if some non-overlapping information between these two SFs could improve ML. Correlations between other features are shown in Table 1. Despite showing statistical significance due to the large sample sizes, correlations were low suggesting that the features are independent of each other and therefore unlikely to contribute redundant information to the ANN. Thus, our benchmark ANN configuration comprised of 35 inputs for which we expected maximal learning outcomes: 7 electrodes provided spatial information across the brainstem, and all 5 SFs provide relevant, independent information about the nerve type and the side of the body from which the DCN response was evoked.

**Table 1:** Pearson’s correlations for input feature pairs.

Correlation	Pearson’s correlation	*p*-value	Significance
HF integral vs. HF peak count	0.89	<2.2e-16	^∗∗∗^
HF integral vs. P1N1 amplitude	0.49	<2.2e-16	^∗∗∗^
P1N1 amplitude vs. HF peak count	0.46	<2.2e-16	^∗∗∗^
P1N1 amplitude vs. N1 latency	0.17	<2.2e-16	^∗∗∗^
N1 latency vs. P1N1 slope	0.07	<2.2e-16	^∗∗∗^
HF integral vs. N1 latency	0.05	<2.2e-16	^∗∗∗^
HF peak count vs. N1 latency	0.04	1.60e-13	^∗∗∗^
P1N1 amplitude vs. P1N1 slope	0.03	1.62e-10	^∗∗∗^
HF peak count vs. P1N1 slope	0.01	0.03959	^∗^
HF integral vs. P1N1 slope	−0.005	0.294	

*^∗^p < 0.05, ^∗∗∗^p < 0.001.*

**FIGURE 2:**
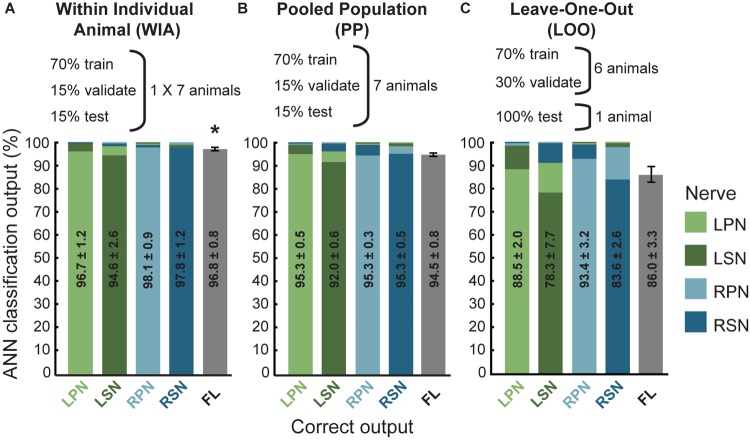
Artificial neural network classification of nerve origin from multi-electrode array surface recordings – three data partitioning approaches. ANN prediction accuracies are shown using three approaches to divide data sets (data partitioning approaches shown above each bar graph). For each correct target output (indicated on the abscissa, nerves color coded according to the figure key) the mean classifications are shown as bars colored for each nerve (color coded along the ordinate, stacked in order of classification magnitudes). Mean ± SEM indicated for correct classifications. Ideal learning (100% classification accuracy) would see the bar colors match their abscissa label. **(A)** The Within Individual Animal (WIA) approach divided responses from individual animals into training, validation, and testing data sets (data from mean of 7 animals shown). **(B)** For the Pooled Population (PP) approach, responses from all 7 animals were pooled together into a single data set before sub-dividing into training, validation, and testing data sets (data from mean of 10 learning cycles); **(C)** the Leave-One-Out (LOO) approach trained and validated using data pooled from 6 animals and tested the prediction on the remaining animal (data from mean of 7 animals). For all three approaches, machine learning performance was obtained from the average over 10 learning cycles (algorithm initiated 10 times with random starting conditions). Feature-learnability is derived as the mean and SEM of all correct classifications as is represented by the gray bars in the right-hand column of each graph (feature-learnability benchmark indicated by the asterisk in **A**). ANN, artificial neural network; LOO, leave-one-out; LPN, left peroneal nerve; LSN, left sural nerve; FL, feature-learnability; PP, pooled population; RPN, right peroneal nerve; RSN, right sural nerve; WIA, within individual animal.

Three different approaches for the training, validation and testing data sets from 7 animals were applied to the ANN configuration (WIA, PP, and LOO, see section “Materials and Methods”). The machine-learning outcomes from three approaches are shown in Figure 2 and a summary of the statistical comparisons performed in the remainder of this section are presented in Table 2. All classifications were well beyond chance performance (25%) for all three approaches. Feature-learnability using the WIA approach (benchmark configuration) was 96.8 ± 0.8% (mean ± SEM, Figure 2A). The most accurately classified nerve evoked signals originating from RPN stimulation (98.1 ± 0.9%). The greatest error was found in LSN evoked signals, which were sometimes falsely classified as LPN evoked signals (3.6 ± 1.7% error). There were no differences in the classification of side (Table 2, row A), nerve type (Table 2, row B), bilateral nerve pairs (Table 2, rows C, D), or between the different ipsilateral nerves (Table 2, rows E, F).

**Table 2:** Statistical comparisons for benchmark feature-learnability and ML approaches (Figure 2).

	Statistical comparison	*p*-value	Significance	Test	Figure
A	WIA Side (left vs. right)	0.167		Two-way ANOVA	2A
B	WIA Nerve (sural vs. peroneal)	0.474		Two-way ANOVA	2A
C	WIA Peroneal bilateral nerve pair (LPN vs. RPN)	0.921		Two-way ANOVA, Tukey HSD *post hoc*	2A
D	WIA Sural bilateral nerve pair (LSN vs. RSN)	0.521		Two-way ANOVA, Tukey HSD *post hoc*	2A
E	WIA Different ipsilateral nerve (LPN vs. LSN)	0.81		Two-way ANOVA, Tukey HSD *post hoc*	2A
F	WIA Different ipsilateral nerve (RPN vs. RSN)	0.999		Two-way ANOVA, Tukey HSD *post hoc*	2A
G	WIA vs. PP	0.7		One-way ANOVA, Tukey HSD *post hoc*	2A,B
H	WIA vs. LOO	0.023	^∗^	One-way ANOVA, Tukey HSD *post hoc*	2A,C
I	PP vs. LOO	0.066	•	One-way ANOVA, Tukey HSD *post hoc*	2B,C

*LOO, leave-one-out; LPN, left peroneal nerve; LSN, left sural nerve; PP, pooled population; RPN, right peroneal nerve; RSN, right sural nerve; WIA, within individual animal. ^•^p < 0.1, ^∗^p < 0.05.*

Feature learnability using the PP approach was 94.5 ± 0.8% (Figure 2B) which was not significantly different to the benchmark configuration (Table 2, row G). The most accurately predicted nerve evoked signal was from the RSN (95.4 ± 0.5%), whilst the greatest error occurred from LSN-evoked signals being falsely classified as LPN-evoked (4.5 ± 0.4% error).

Feature learnability using the LOO approach was 86.0 ± 3.3% (Figure 2C) which was significantly reduced compared to the WIA approach (Table 2, row H), but failed to reach a significant difference compared to the PP approach (Table 2, row I). The most accurately predicted nerve evoked signals were from RPN (93.4 ± 3.2%) and the greatest errors occurred with RSN-evoked signals being falsely classified as RPN-evoked (14.3 ± 2.6% error).

Overall, these results demonstrate that DCN responses contain a combination of SFs expressed over the seven electrodes that are unique to the nerve from which they are evoked. The ability of the ANN to learn from examples taken from one population of animals and classify data obtained from an independent set of animals, indicates that the SFs are robustly conserved and can be generalized across different animals.

### How do Individual Electrode Positions Contribute to Feature-Learnability?

**FIGURE 3:**
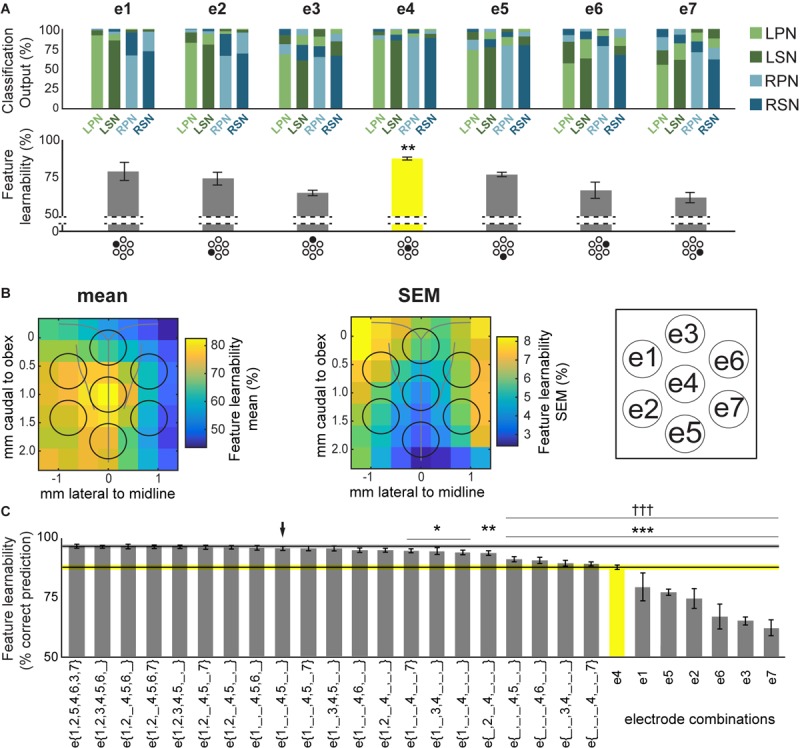
Feature-learnability of electrode surface recordings. **(A)** Top panel shows the mean ANN classification outputs for each nerve, from inputs derived from all 5 SFs at individual sMEA electrodes using the WIA approach (Figure 2A). Feature-learnability, i.e., the mean correct prediction accuracy of all 4 nerve outputs, is shown in the bar graph below each ANN output. Each electrode is indicated under its respective bar graph by the black-shaded dot in the sMEA schematic representation (see insert for electrode labels). Asterisks indicate that e4 demonstrated feature-learnability that was significantly higher than all other electrodes (yellow bar). **(B)** Relative mean feature-learnability (left panel) and SEM (middle panel), derived from individual electrodes (e1 to e7, as per insert) from seven animals, are color coded onto normalized maps of the brainstem surface. Black circles indicate electrode positions (see insert for electrode labels). **(C)** Feature-learnability for electrode combinations are represented from highest (i.e., the benchmark) to lowest ranking (left to right). Single electrode feature-learnability [as in the bottom panel of **(A)**] are also ranked to facilitate comparisons with other electrode combinations. Black horizontal line indicates the mean feature-learnability of e{1–7} (gray ghosting indicates ± SEM) or e4 (yellow, ghosting indicates ± SEM). Black arrow indicates the highest-ranked 3-electrode combination. Asterisks indicate significant difference from benchmark (WIA approach); daggers represent significant difference from arrow. e1, electrode 1; e2, electrode 2; e3, electrode 3; e4, electrode 4; e5, electrode 5; e6, electrode 6; e7, electrode 7; e{1–7}, electrodes 1 to 7 inclusive; LPN, left peroneal nerve; LSN, left sural nerve; RPN, right peroneal nerve; RSN, right sural nerve; SF, signal feature; WIA, within individual animal (see section “Materials and Methods”). ^∗^*p* < 0.05, ^∗∗^*p* < 0.01, ^∗∗∗^*p* < 0.001, ^†††^*p* < 0.001, LMEM.

To rank electrode/s positions in order of their capacity to report the most relevant information for determining the origin of sensory input, we compared the feature-learnability from all 5 SFs extracted from individual electrodes to the feature-learnability benchmark (i.e., data from all SFs across e{1–7}). In all cases, feature-learnability was significantly greater than chance levels (25%). A summary of the results of statistical testing in Section “How do Individual Electrode Positions Contribute to Feature-Learnability?” and “How do Subsets of Electrode Positions Contribute to Feature-Learnability?” are presented in Table 3. Overall, there was a significant difference of feature-learnability among electrode positions (Table 3, row A), but no significant effect of which nerve was activated (Table 3, row B). The highest feature-learnability achieved from a single electrode position with all 5 SFs was 87.7 ± 1.0% from e4, and the lowest was 62.1 ± 3.4% from e7 (Figure 3A).

**Table 3:** Statistical comparisons of electrode positions (Figure 3).

	Statistical comparison	*p*-value	Significance	Test	Figure
A	Difference between electrode positions (electrode effect)	2.20E-15	^∗∗∗^	LMEM	3A
B	Difference between nerves (nerve effect)	0.83		LMEM	3A
C	e4 vs. each individual electrode	≤0.011	^∗^	LMEM, FDR *post hoc*	3C
D	e4 best nerve vs. worst nerve (RPN vs. LSN)	0.27		Paired *t*-test	3A
E	Benchmark vs. individual electrodes	<2e-16	^∗∗∗^	LMEM, FDR *post hoc*	3C
F	Rostral left vs. right electrodes (e1 vs. e6)	<2e-4	^∗∗∗^	LMEM, FDR *post hoc*	3C
G	Caudal left vs. right electrodes (e2 vs. e7)	<2e-4	^∗∗∗^	LMEM, FDR *post hoc*	3C
H	Rostral vs. caudal side electrodes (e{1,6} vs. e{2,7})	0.046	^∗^	LMEM, FDR *post hoc*	3C
I	Left electrodes (e1 vs. e2)	0.153		LMEM, FDR *post hoc*	3C
J	Right electrodes (e6 vs. e7)	0.153		LMEM, FDR *post hoc*	3C
K	Middle rostral vs. caudal electrodes (e3 vs. e5)	2.00E-04	^∗∗∗^	LMEM, FDR *post hoc*	3C
L	e4 vs. e{1,4}	0.012	^∗^	LMEM, FDR *post hoc*	3C
M	e4 vs. e{2,4}	0.014	^∗^	LMEM, FDR *post hoc*	3C
N	e4 vs. e{4,5}	0.023	^∗^	LMEM, FDR *post hoc*	3C
O	e4 vs. e{4,6}	0.017	^∗^	LMEM, FDR *post hoc*	3C
P	e4 vs. e{3,4}	≥0.215		LMEM, FDR *post hoc*	3C
Q	e4 vs. e{4,7}	≥0.215		LMEM, FDR *post hoc*	3C
R	e{1,4} vs. all other electrode pairs except e{2,4}	≤0.021	^∗^	LMEM, FDR *post hoc*	3C
S	e{1,4} vs. e{2,4}	0.699		LMEM, FDR *post hoc*	3C
T	Benchmark vs. each electrode pair	≤0.017	^∗^	LMEM, FDR *post hoc*	3C
U	e{1,4,5} vs. e{1,4}, e{2,4}	≥0.161		LMEM, FDR *post hoc*	3C
V	Benchmark vs. e{1,4,5}, e{1,2,4}, e{1,4,6}	≥0.395		LMEM, FDR *post hoc*	3C
W	Benchmark vs. e{1,3,4}, e{1,4,7}	≤0.047	^∗^	LMEM, FDR *post hoc*	3C
X	e{1,4,5} vs. all individual electrodes and electrode pairs, except e{1,4}, e{2,4}	<2e-16	^∗∗∗^	LMEM, FDR *post hoc*	3C

*e1, electrode 1; e2, electrode 2; e3, electrode 3; e4, electrode 4; e5, electrode 5; e6, electrode 6; e7, electrode 7; e{1–7}, electrodes 1 to 7 inclusive; FDR, false-discovery rate; LMEM, linear mixed-effects model; See Table 2 for other abbreviations. ^∗^p < 0.05, ^∗∗∗^p < 0.001.*

To view the spatial arrangement of feature-learnability across individual electrode positions, an interpolated map of mean and SEM feature-learnability was constructed from the seven animals (Figure 3B). The maps illustrate that the region occupied by e4 facilitates the greatest mean learnability with the lowest variability (SEM) across the DCN surface, followed by 2 adjacent regions: below e4 on the midline between e4 and e5; and to the left of e4, equidistant between e1, e2 and e4. Inspection of learnability maps from individual animals revealed that while all animals had a learnability hotspot located at the e4 position, the shoulder of the hotspot extended in seemingly random directions toward e1, e2, and e5 or a combination of two of these positions. Most animals displayed reduced learnability at the e3, e6, and e7 positions. *Post hoc* analysis confirmed that information acquired from e4 positions was significantly better at classifying nerve origin than all the other individual electrode positions (Table 3, row C; yellow bar, Figure 3C). Furthermore, despite being a midline electrode, information acquired from e4 predicted the correct side of the body from which responses were evoked with surprisingly high accuracy (92.6 ± 0.6%; chance level = 50%). Of responses from e4, RPN inputs were most accurately classified (89.8 ± 3.3%) whilst LSN responses resulted in the lowest mean classification accuracy (85.3 ± 4.4%; Figure 3A), however, the difference was not significant (Table 3, row D).

Compared to the feature-learnability benchmark configuration, all single electrodes significantly under-performed (Table 3, row E) at classifying the nerve from which a response was evoked (Figure 3C). Of the lateral electrodes, positions occupied by individual left electrodes facilitated higher feature-learnability than right electrode positions for both the rostral (Table 3, row F), and the caudal (Table 3, row G) pairs. These rostral electrodes (e1 and e6) collectively demonstrated greater feature-learnability, on average, compared to the caudal electrodes (e2 and e7, Table 3, row H), while there being no individual significant differences detected on either side (Table 3, rows I, J). The most caudal electrode on the midline (e5) achieved a mean learnability that was significantly greater than the most rostral midline electrode (e3, Table 3, row K).

In summary, these analyses indicate that feature-learnability of 5 SFs from any individual electrode is: (1) less than learnability obtained from 5 SFs across all 7 electrodes, and (2) generally maximum for e4 positions, but with shoulders that appear to protrude randomly toward e1, e2, and e5, or a combination of these, in different animals. Moreover, feature-learnability is asymmetrically represented across the surface of the DCN with an overall bias toward the left side.

### How do Subsets of Electrode Positions Contribute to Feature-Learnability?

We aimed to determine the minimum number, and unique electrode positions to achieve near-benchmark feature-learnability. As e4 had the highest prediction accuracy of any individual electrode, we incrementally added blocks of 5 SFs derived from other single electrodes. Any improvement in feature-learnability would indicate the addition of relevant, but not redundant, information.

A summary of feature-learnability rank order from various electrode combinations is shown in Figure 3C. Starting with e4 (yellow bar, Figure 3C), feature-learnability was significantly improved by adding e1, e2, e5, or e6, but not e3, or e7 (Table 3, rows L–Q, respectively). The best performing electrode pair was e{1, 4}, which demonstrated feature-learnability significantly greater than four of the six electrode pair combinations (94.2 ± 0.9%, Table 3, row R) with the exception of e{2, 4} (Table 3, row S). None of the electrode pair combinations achieved near-benchmark feature-learnability (Table 3, row T). These findings demonstrate that, of the electrode pairs, positions occupied by e4 plus one of the left electrodes, e1 or e2, were best for classifying nerve inputs.

As none of the electrode pair combinations achieved near-benchmark feature-learnability, we continued to add inputs from individual electrodes to the highest performing electrode pair (e{1, 4}). The combination with the highest feature-learnability was with the addition of e5 to e{1, 4} (95.9 ± 0.9%; black arrow Figure 3C). While failing to be significantly different to e{1, 4}, or e{2, 4} (Table 3, row U), e{1, 4, 5}, along with e{1, 2, 4} and e{1, 4, 6} all achieved near-benchmark feature-learnability (Table 3, row V). The other two combinations e{1, 3, 4} and e{1, 4, 7} were both significantly lower than the benchmark (Table 3, row W), indicating that e3 and e7 contributed the least useful information when added to e{1, 4}. Aside from e{1, 4} and e{2, 4}, e{1, 4, 5} significantly outperformed the other electrode pairs and all individual electrodes (Table 3, row X). These observations were consistent with the feature-learnability map that showed learnability was maximal at e4 positions, but reduced closest to e3, e6, and e7 (Figure 3B).

In summary, although all individual electrodes classified significantly above chance levels, the best performing individual electrode was e4. The most efficient electrode combination was e{1, 4, 5}, which could classify the nerve from which a response was evoked with an accuracy that was not significantly different to the benchmark.

### How do Signal Features Contribute to Feature-Learnability?

**FIGURE 4:**
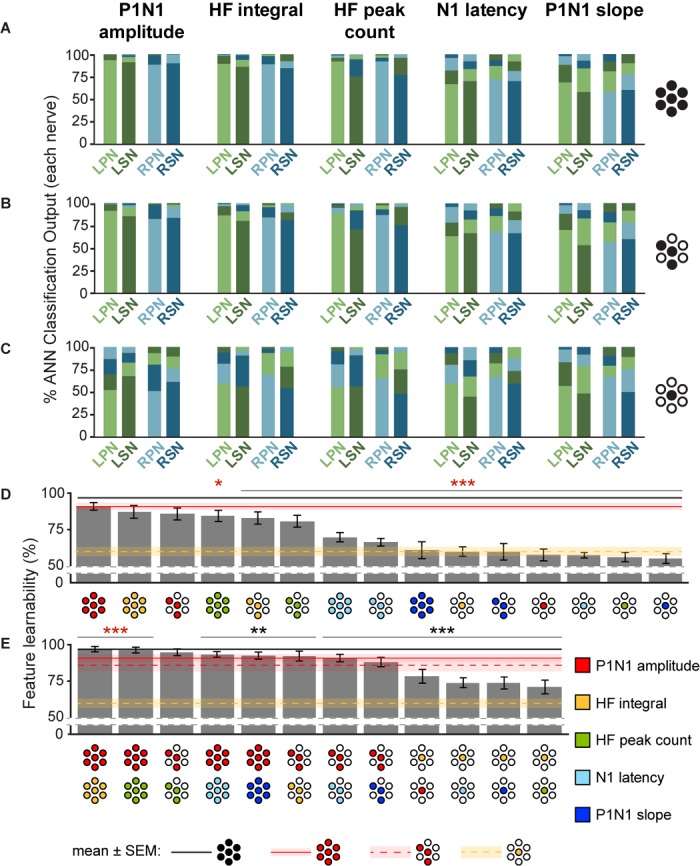
Feature-learnability of individual, and combinations of signal features generated from all electrodes, best three electrodes, and best single electrode. The mean classification accuracy for each nerve using the WIA approach (Figure 2A) generated from individual SFs are shown when data was quantified from: **(A)** all electrodes (e{1–7}), **(B)** the top three performing electrodes (e{1, 4, 5}) and **(C)** best electrode (e4). Schematics to the right of graphs show the electrodes from which SFs were quantified (as per Figure 3B insert). **(D)** Feature-learnability for each SF over combinations of electrodes (all, e{1–7}; best 3, e{1, 4, 5}; best 1, e4) are ranked from highest to lowest (left to right). Schematics for each bar indicate the electrodes (filled circles, as per Figure 3B insert) from which SFs were quantified (colors indicate respective SF as per the figure key). **(E)** Same as **(D)**, but each bar represents the mean feature-learnability achieved using the best single SF for each electrode combination, with the addition of one of each of the other SFs. Black horizontal line with gray ghosting indicates the mean ± SEM feature-learnability of the benchmark (e{1–7}); red lines and ghosting indicates mean ± SEM feature-learnability of P1N1 amplitude/e{1, 4, 5} (dashed) and P1N1 amplitude/e{1–7} (solid); yellow-dashed lines and ghosting indicates mean ± SEM feature-learnability of HF integral/e4 alone; black asterisks indicate feature-learnability significantly lower than the benchmark (e{1–7}) and red asterisks indicate feature-learnability significantly better than P1N1 amplitude/e{1–7}. Abbreviations as per Figure 3.

To determine which DCN SFs contributed the most information to facilitate accurate nerve prediction, we investigated the feature-learnability of individual SFs across all seven electrodes (e{1–7}, Figure 4A), the best three-electrode combination (e{1, 4, 5}, Figure 4B) and the best single (e4, Figure 4C) electrode. In all cases, feature-learnability was significantly greater than chance levels (25%). When single SFs were examined across e{1–7}, P1N1 amplitude had the highest prediction accuracy (90.8 ± 2.7%) followed by HF integral, HF peak count, N1 latency and P1N1 slope; Figure 4D). Feature-learnability derived from all of the single SFs tested across e{1–7} was significantly lower than the benchmark (Table 4, row A). Interestingly, the same feature-learnability rank order was observed when each SF was tested across e{1, 4, 5}. However, the rank order was no longer conserved when tested across e4 alone; HF integral achieved the highest-ranked feature-learnability for e4, with P1N1 amplitude ranked second (Figure 4D). Overall, P1N1 amplitude/e{1, 4, 5} significantly outperformed two of the e{1–7} single feature combinations: N1 latency/e{1–7} and P1N1 slope/e{1–7} (Table 4, row B). P1N1 amplitude/e{1, 4, 5} also achieved higher ranking than HF peak count/e{1–7}, although feature-learnability was not significantly different (Table 4, row C).

**Table 4:** Statistical comparisons of feature-learnability of signal features (Figure 4).

	Statistical comparison	*p*-value	Significance	Test	Figure
A	Benchmark vs. individual SFs extracted from e{1–7}	≤5e-4	^∗∗∗^	LMEM, FDR *post hoc*	4D
B	P1N1 amplitude/e{1,4,5} vs. N1 latency/e{1–7}, P1N1 slope/e{1–7}	<2e-16	^∗∗∗^	LMEM, FDR *post hoc*	4D
C	P1N1 amplitude/e{1,4,5} vs. HF peak count/e{1–7}	0.671		LMEM, FDR *post hoc*	4D
D	Benchmark vs. P1N1 amplitude + HF integral/e{1–7}	0.773		LMEM, FDR *post hoc*	4E
E	P1N1 amplitude/e{1–7} vs. P1N1 amplitude + HF integral/e{1–7}	<2e-16	^∗∗∗^	LMEM, FDR *post hoc*	4E
F	P1N1 amplitude/e{1–7} vs. P1N1 amplitude + HF peak count/e{1–7}	<9e-4	^∗∗∗^	LMEM, FDR *post hoc*	4E
G	P1N1 amplitude/e{1–7} vs. P1N1 amplitude + N1 latency/e{1–7}	0.394		LMEM, FDR *post hoc*	4E
H	P1N1 amplitude/e{1–7} vs. P1N1 amplitude + P1N1 slope/e{1–7}	0.494		LMEM, FDR *post hoc*	4E
I	P1N1 amplitude/e{1,4,5} vs. P1N1 amplitude + HF integral/e{1,4,5}	<2e-16	^∗∗∗^	LMEM, FDR *post hoc*	4E
J	P1N1 amplitude/e{1,4,5} vs. P1N1 amplitude + HF peak count/e{1,4,5}	0.006	^∗∗^	LMEM, FDR *post hoc*	4E
K	P1N1 amplitude/e{1,4,5} vs. P1N1 amplitude + N1 latency/e{1,4,5}	0.078		LMEM, FDR *post hoc*	4E
L	P1N1 amplitude/e{1,4,5} vs. P1N1 amplitude + P1N1 slope/e{1,4,5}	0.552		LMEM, FDR *post hoc*	4E
M	Benchmark vs. P1N1 amplitude + HF integral/e{1,4,5}	0.058		LMEM, FDR *post hoc*	4E
N	Benchmark vs. SF pairs from e{1–7}	≤3e-4	^∗∗∗^	LMEM, FDR *post hoc*	4E
O	HF integral/e4 vs. each HF integral/e4 with the addition of each of the other SFs/e4	0.007	**	LMEM, FDR *post hoc*	4E
P	Benchmark vs. SF pairs from e4	<2e-16	^∗∗∗^	LMEM, FDR *post hoc*	4E

*SF, signal features. For all other abbreviations**s**ee Tables 2, 3 for abbreviations. ^∗∗^p < 0.01, ^∗∗∗^p < 0.001.*

We wanted to determine which of the other SFs would improve feature-learnability if added to the best SF for each arrangement. We therefore quantified feature-learnability with P1N1 amplitude (e{1–7} and e{1, 4, 5}) and HF integral (e4) in combination with each of the remaining four SFs (Figure 4E). Of these combinations, P1N1 amplitude/e{1–7} combined with HF integral/e{1–7} had the highest feature-learnability (96.7 ± 1.9%), which was not significantly different to the benchmark (Table 4, row D). P1N1 amplitude/e{1–7} feature-learnability was significantly improved by the addition of HF integral/e{1–7} (Table 4, row E) and HF peak count/e{1–7} (Table 4, row F), but not N1 latency/e{1–7} (Table 4, row G) or P1N1 slope/e{1–7} (Table 4, row H and Figure 4E). This was also the case for P1N1 amplitude/e{1, 4, 5} feature-learnability, which was significantly improved by the addition of HF peak count/e{1, 4, 5} (Table 4, row I) and HF integral/e{1, 4, 5} (Table 4, row J), but improvement from adding N1 latency/e{1, 4, 5} or P1N1 slope/e{1, 4, 5} failed to reach significance (Table 4, rows K, L and Figure 4E). Of the e{1, 4, 5} combinations, only P1N1 amplitude + HF peak count/e{1, 4, 5} was able to achieve near-benchmark feature-learnability (Table 4, row M), while the remaining combinations demonstrated feature-learnability significantly lower than the benchmark (Table 4, row N). This demonstrates that feature-learnability from 6 inputs (2 SFs acquired from 3 electrodes) was not significantly different to that obtained from 35 inputs (5 SFs acquired from 7 electrodes). Finally, HF integral/e4 feature-learnability was improved by the addition of each of the other SFs (Table 4, row O and Figure 4E). However, feature-learnability from each e4 combination remained significantly lower than the benchmark (Table 4, row P).

In summary, P1N1 amplitude was the best individual SF for feature-learnability of the nerve from which a DCN response was evoked when using 3 or 7 electrodes. HF integral achieved the highest feature-learnability ranking from e4 alone, although each SF performed significantly greater than chance on this electrode. When limited to only two SFs, P1N1 amplitude + HF integral/e{1–7} achieved the highest feature-learnability of all the SF/electrode combinations, however, only 6 inputs (P1N1 amplitude + HF peak count/e{1, 4, 5}) were required to reach near-benchmark feature-learnability.

### Maximizing Feature-Learnability With Minimal Inputs

**FIGURE 5:**
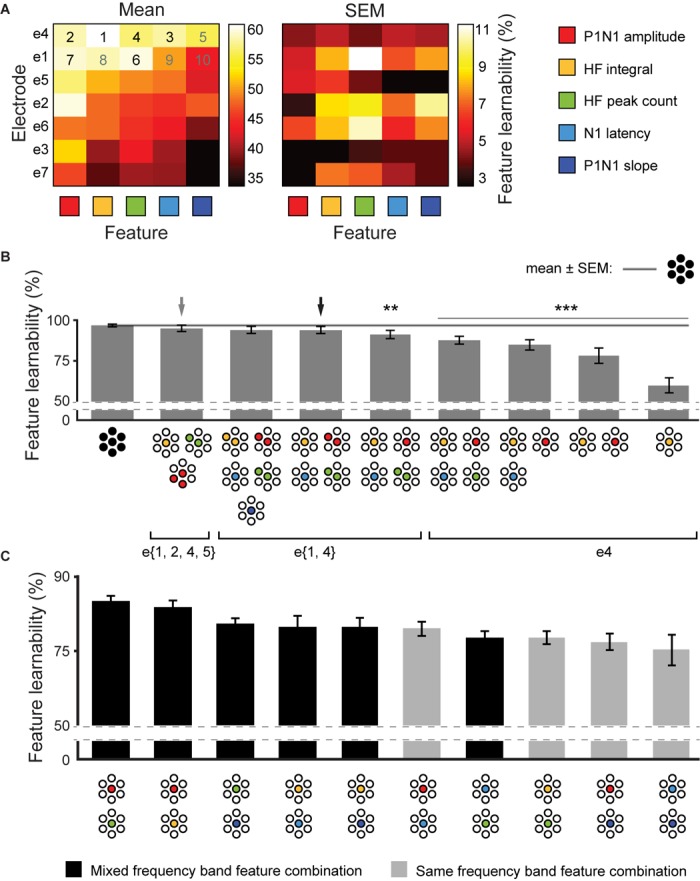
Achieving near-benchmark feature-learnability with minimal SF/electrode combinations. **(A)** Shows mean (left panel) and SEM (right panel) feature-learnability for each SF/electrode combination; heat map color coded between maximum (white) and minimum (black) feature-learnability. Rows are ordered from highest- (top) to lowest- (bottom) ranked electrodes, and columns are ordered from highest- (left) to lowest- (right) ranked SF. Numbers in the top two rows indicate the order of next-best SF/electrode combination, where back numbers indicate those incorporated to achieve near-benchmark ANN according to the electrode minimization strategy (see section “Materials and Methods”). **(B)** Selected feature-learnability ranks determined by systematically adding the next best performing SFs/electrode combination to HF integral/e4, until feature-learnability was not significantly different to the benchmark (see section “Materials and Methods”). Black arrow indicates the SF/electrode combination with the highest feature-learnability and fewest inputs when minimizing total number of electrodes was the priority. Gray arrow indicates the SF/electrode combination with the highest feature-learnability and fewest inputs when minimizing absolute input number was the priority. Black horizontal line with gray ghosting indicates the benchmark ± SEM feature-learnability. Black asterisks indicate feature-learnability significantly lower than the benchmark. **(C)** Feature-learnability ranks determined by combining each SF pair acquired from e4. Feature combinations that included only HF features or only LF features (light gray bars) generally ranked lower than combinations containing pairs of one HF and one LF feature (black bars). Abbreviations as per Figure 3.

We established that feature-learnability continued to perform well above chance after reducing the number of electrodes (Figure 3) or SFs (Figure 4). Here, we aimed to find SF/electrode combinations that achieve near-benchmark learnability by systematically incorporating the next-best ranked electrode/SF configuration as inputs into an ANN. Mean and SEM feature-learnability for each individual SF/electrode combination is shown in Figure 5A. HF integral/e4 achieved the highest feature-learnability (60.1 ± 4.6%) of all the individual SF/electrode combinations, while P1N1 slope/e7 achieved the lowest (33.9 ± 3.5%), although it remained significantly above chance levels (25%).

We used an adapted SFS algorithm (see section “Materials and Methods”) for two input minimization strategies to subsequently incorporate the next-best SF/electrode combinations to HF integral/e4 for feature-learnability. The first strategy aimed to minimize the number of inputs required to achieve near-benchmark feature-learnability, irrespective of the electrodes from which they were acquired. Thus, the next-best SF/electrode inputs, according to Figure 5A, were selected without restricting them to specific electrodes. We found that 6 inputs acquired from 4 electrodes and three SFs (e{1, 2, 4, 5}), achieved near-benchmark feature-learnability (95.0 ± 1.9%; *p* = 0.067, paired *t*-test, gray arrow, Figure 5B). The second strategy prioritized minimizing the number of electrodes used to acquire input features, thus next-best features, according to Figure 5A were restricted to the same electrode as the previous SF/electrode combination until exhausting all possibilities for that electrode. We found that 2 electrodes: e{1, 4} (4 SFs acquired from e4 and 2 SFs from e1, black arrow, Figure 5B) achieved a feature-learnability of 94.0 ± 2.2%, which failed to reach a significant difference from the benchmark (*p* = 0.06, paired *t*-test). We continued to add the remaining inputs from these 2 electrodes (i.e., N1 latency and P1N1 slope), however, although these two additional inputs improved the learnability to 94.9 ± 1.9%, it failed to significantly improve feature-learnability from the 6 inputs across e{1, 4} (*p* = 0.094, paired *t*-test). The first and second strategies yielded the same minimum number of inputs, and there was no significant difference in their feature-learnability (*p* = 0.225, paired *t*-test), however, the first approach was ranked higher.

The electrode minimization strategy required 2 electrodes (10 possible inputs). The sum of possible combinations using 3, 4, 5, and 6 SF/electrode inputs is 792. We therefore tested all 792 possible combinations, to confirm that the second strategy yielded a result that was not significantly different to the best possible outcome. This exhaustive approach also yielded 6 inputs: two electrodes (e{1, 4}) and three SFs (P1N1 amplitude, N1 latency and HF peak count), with a feature-learnability (94.2 ± 2.2%), not significantly different to the second approach (*p* = 0.62, paired *t*-test). It was not feasible to test all possible feature/electrode combinations of the first method as the sum of possible 3, 4, 5, and 6- feature/electrode combinations, from 35 possible inputs, is over 2 million.

Across all possible combinations of two SFs acquired from e4 (Figure 5C), those with one HF and one LF feature yielded significantly greater learnability than those combinations with two HF or two LF features (*p* = 0.006, LMER). The best performing pairs were P1N1 amplitude with HF peak count (highest ranked) and P1N1 amplitude with HF integral (ranked second).

In summary, 6 inputs were sufficient to achieve near-benchmark feature-learnability, which arose from either 4 electrodes (e{1, 2, 4, 5}) with 3 SFs (P1N1 amplitude, HF integral, and HF peak count; gray arrow, Figure 5B), or 2 electrodes (e{1, 4}) with 4 SFs (P1N1 amplitude, HF integral, HF peak count, N1 latency; black arrow, Figure 5B); there was no significant difference between these strategies, although the first input minimization strategies ranked higher. Maximum feature-learnability of two inputs from e4 was achieved by combining a LF and HF feature (Figure 5C).

### Signal Feature Asymmetry

Feature-learnability was highest among individual electrodes when data was acquired from e4, a midline electrode (Figure 3C). This raises the question: how could the ANN learning algorithm discriminate between DCN signals evoked from bilateral nerve pairs that are recorded from the same midline electrode? The most parsimonious explanation is that left sided nerves evoke different DCN responses to right sided nerves. We therefore set out to determine if left/right prediction accuracy of midline electrodes truly represents the asymmetric expression of SFs, or rather, if this observation resulted from misalignment of the sMEA over the brainstem midline.

#### sMEA Position Asymmetry

Signal feature asymmetries could result if the recording sMEA is not adequately aligned along the midline, resulting in recording larger signals when stimulating a nerve on one side compared to the other. If the middle electrodes were arranged directly over the brainstem midline, the distance of the medial edge of both left and right electrodes would measure 470 μm from the midline (Supplementary Figure S1A). We found that the mean distance from the brainstem midline to the left electrodes was 479 ± 101 μm, and 453 ± 101 μm to the right electrodes, indicating that on average there was a small shift to the left of approximately 13 μm. Electrode shift is indicated for each animal in Supplementary Figure S1A. Of the 7 animals, 5 had electrodes shifted less than one-quarter of an electrode diameter from the midline on either side (electrode diameter = 700 μm); the remaining 2 animals, the sMEA was shifted just less than two-thirds of an electrode diameter on either the left (A2, −417 μm) or right (A5, +430 μm) sides.

#### DCN Surface Activity Asymmetry

If electrode shift significantly impacted SFs, we would expect to find responses recorded from left-shifted sMEAs to be consistently biased in magnitude and opposite to responses recorded from right-shifted sMEAs. For each bilateral nerve pair, we therefore determined the side of the body for which each SF was significantly greater at the central electrode (e4 side dominance). Supplementary Figure S1B displays the side dominance (L or R) for each animal and SF in order of electrode position. The animals with the most right-shifted sMEAs (A5 and A7) displayed right-side dominance for all SFs except for N1 latency (which signifies shorter right-sided latencies). However, this was also the case for the animals with the most left-shifted sMEAs (A2 and A4), indicating that electrode shift was unlikely to be responsible for the SF asymmetry.

Next, we quantified the ML error (100 – ML accuracy) associated with each bilateral nerve pair (calculated as the mean ML error of both nerves for each bilateral nerve pair at e4, color coded, Supplementary Figure S1B). Across all animals and SFs (i.e., 35 instances from 5 SFs for 7 animals), there were 26/35 instances (74%) where both bilateral nerve pairs displayed a side dominance for a SF (Supplementary Figure S1B). 3/26 of these instances (12%) had at least one nerve pair producing errors at or above chance (indicated by one or two asterisks, top right corner of boxes, Supplementary Figure S1B; see: HF peak count, A5 and A3; P1N1, slope A2). The remaining 9 instances had either one (7/35) or both (2/35) nerve pairs that failed to demonstrate a side dominance. In all 9 instances (100%), at least one nerve pair performed at or above the chance of producing an error (at least one asterisk, Supplementary Figure S1B). Thus, failure to display a SF side dominance in at least one nerve pair was significantly associated with the e4 mean ML error occurring above chance levels (*p* = 1.44e-6, Pearson’s Chi-squared test).

We were curious to see if the side dominance observation made for e4 could be generalized across all electrodes. For each animal, we summed the SF values across all 7 electrodes to determine the TRM (see Supplementary Figure S1C) for a given SF. We then established if there was a significant difference in the TRM when stimulating bilateral nerve pairs from each side of the body (i.e., significant TRM side dominance as indicated by L and R, Supplementary Figure S1C). We observed a strong and significant relationship (Pearson’s correlation = 0.995, *p* = 0.0005) between the number of side-dominant bilateral nerve pairs (i.e., number of black or gray squares, Supplementary Figure S1C) for each SF, and the feature-learnability of each SF (determined from e{1–7}, Figure 4D).

In summary, our data demonstrates that (i) SFs are expressed asymmetrically across the sMEA, (ii) the ML algorithm’s capacity to resolve left/right sides from a central electrode arises from a real difference in the SF magnitudes that are evoked from the different sides and not due to variations of electrode placement, and (iii) the incidence of bilateral nerve pair side dominance across all electrodes is strongly correlated to overall ML prediction accuracy.

### Potential Loci of ANN Classification Errors

**FIGURE 6:**
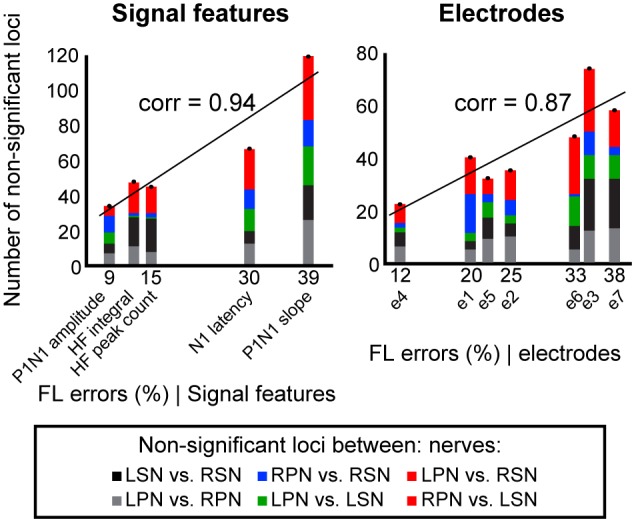
Non-significant loci correlations to feature-learnability. To investigate the relationship between the occurrence of non-significant loci (see section “Materials and Methods” and Supplementary Figure S2) and feature-learnability, we summed the total number of non-significant loci for each SF and electrode. Graphs show non-significant loci quantified and stacked for each SF (left panel), and each electrode (right panel). The *y*-axis represents the total number of non-significant loci, and colors in each column indicate quantities for each type of non-significant loci category (insert, see also Supplementary Figure S2 for distribution of non-significant loci across animals, SFs and electrodes). The *x*-axis represents the feature-learnability (FL) errors (see section “Materials and Methods”). A linear model was constructed to represent the relationship between the number of non-significant loci and feature-learnability errors. Pearson’s correlations (corr) between the number of non-significant loci and feature-learnability errors are shown. These positive relationships demonstrate that feature-learnability errors are proportional to the non-significant loci. FL error, feature-learnability error; see Figure 3 for all other abbreviations.

To demonstrate that feature-learnability is influenced by how similar, or different, the underlying biological events were, we sought to demonstrate that classification errors generated by the ANN were consistent with statistical observations of the input SFs. We hypothesized that the ANN would generate more confusion errors when classifying responses from two inputs that were not significantly different. Non-significant loci (Supplementary Figure S2) were classified (as per insert of Supplementary Figure S2) into bilateral nerve pair errors (indicated by black and gray for sural and peroneal nerves, respectively), right and left sided nerve errors (blue and green, respectively), and opposite nerve + side errors (red). We found a strong positive correlation between feature-learnability errors (100 – feature-learnability) and non-significant loci (Figure 6) when summed across each SF (Pearson’s correlation = 0.94; slope of linear model = 2.51, *R*^2^ = 0.88, *p* = 0.018, LM), or across each electrode (Pearson’s correlation = 0.87; slope = 1.67, *R*^2^ = 0.75, *p* = 0.011).

These findings show that (i) feature-learnability confusion errors are associated with DCN response SFs with non-significant loci, and (ii) feature-learnability performance has a strong and significant correlation to physiologically relevant information, i.e., the number of non-significant loci.

## Discussion

In this study we used ML to show that nerve-evoked SFs extracted from the surface of the DCN are unique to the stimulated peripheral nerves and generalize within and across animals. We were able to maintain a high level of classification accuracy for determining peripheral nerve types and locations from SFs acquired from as little as two electrodes, and we provide further evidence that DCN SFs evoked from bilateral nerve pairs are asymmetrically represented across the DCN surface (Loutit et al., 2017). Finally, we correlated statistical observations from nerve-evoked DCN SFs to feature-learnability, thereby demonstrating that our ML approach is sensitive to differences in SFs from nerves innervating different parts of the body that are known to have different afferent compositions. Together, the findings provide new insight into signal patterns across the DCN surface and here we discuss this in context of the DCN’s potential role in future somatosensory neuroprosthetic research.

### Feature-Learnability of Nerve-Evoked DCN Surface Activity

We used feature-learnability as a measure of information relevance contained within SF for resolving a classification problem as to which nerve evoked a DCN response. We fixed the number of ANN hidden layer neurons when altering the number of inputs, because comparison of feature-learnability requires minimal alterations to the ANN configuration. This is equivalent to attributing zero contribution to ML from the omitted input neurons. We are confident that comparing feature-learnability using this approach is valid because the feature-learnability outcomes were consistent with the expected outcomes based on the observations of non-significant loci (Figure 6).

Recently, we showed several HF and LF SFs recorded from the DCN surface are highly reproducible, within and across rats (Loutit et al., 2017). From that study, we selected five DCN SFs that were anticipated to enable clear discrimination between the different nerve types. While we used time-frequency analysis to inform our choice of SFs in the present study, we did not use features of the spectrograms for feature-learnability. The choice of SF used in the present study can be considered a dimensionality reduction of the information contained in the spectrograms (Figure 1B). Therefore, it is likely that extracting features directly from spectrograms would lead to the identification of nerve origin and location with similar success, but the approach we took is computationally less demanding and can be applied over short time windows.

Feature-learnability optimized for data pooled from all animals (PP approach) did not significantly differ from the case where it was optimized for individual animals (WIA approach). This indicates that consistent patterns of activation across the DCN surface are present across animals in response to peripheral nerve stimulation, despite possible variations in electrode placement. Although classification accuracy from the LOO approach was reduced compared to the benchmark, it was not significantly different to the PP approach, remaining more than threefold above chance levels, further demonstrating that SF similarity was apparent across animals. The LOO approach is the most conservative of the three approaches, because there are no samples from the animal used for ANN testing being presented to the ANN for learning. For each animal tested, the data allocated to training and validation in the LOO approach overlapped with the WIA and PP approaches by 86% (i.e., was derived from 6/7 animals). This overlapping learning background enables a meaningful comparison of feature-learnability for each animal tested. Thus, the reduction of feature-learnability of approximately 10% for the LOO versus the WIA and PP approaches can be attributed to the uniqueness of features presented by individual animals. Despite the high feature-learnability in all three approaches, it should be noted that throughout this study peripheral nerves were fully recruited through supramaximal stimulation. We are currently performing a series of experiments that apply natural mechanical stimuli to activate subsets of afferents. Our preliminary data from these studies confirm feature-learnability remains significantly greater than chance levels when the nerve is submaximally stimulated.

The LOO approach demonstrated larger errors associated with confusing ipsilateral nerves rather than confusing side, indicating that compared to the benchmark, reduced learning was associated with individual variations in SFs important for resolving nerve type, but not side. Nevertheless, the prediction accuracy from the conservative LOO approach demonstrates considerable consistency among the selected SFs in their representation across the electrodes for all animals, and is consistent with our previous study which showed that sural and peroneal nerves evoke distinct SFs that are highly conserved across different animals (Loutit et al., 2017).

### DCN Feature-Learnability Mapping

Neural activity, responding to peripheral stimulation, varies as a function of location across the DCN surface (Loutit et al., 2017). Each electrode of the sMEA therefore captures peripheral nerve-evoked DCN electrical signatures from a unique perspective according to its position on the brainstem surface. The quantification of feature-learnability, as a function of location across the DCN, enables the visualization of how information-rich sMEA-acquired SFs are for determining the peripheral source of sensory input. Our map in Figure 3B reveals that, on average, the locations with maximum feature-learnability were most often at e4, followed by e1. The underlying neural elements responsible for generating the SFs at these locations must be located in close proximity, i.e., directly underneath, or next to, the recording electrode, because the recorded activity becomes less informative as distance between the recording electrode and current source increases (Buzsáki et al., 2012). As each electrode of the sMEA has a diameter of 700 μm, the territory covered by e4 corresponds to the extent of hindlimb afferent terminal fields (Maslany et al., 1991), where rat hindlimb receptive fields have been shown to be present at 100 μm below the brainstem surface (Li et al., 2012).

Comparison of the feature-learnability map in the present study, with P1N1 amplitude and HF integral DCN activity maps from our earlier study (Loutit et al., 2017), reveals that the location of 6 out of 10 activity hotspots from the earlier study coincide with the area covered by e4 of the present work. In that study different DCN positions were recorded at different times, but collectively sampled the surface more densely in space. This degree of overlap with P1N1 amplitude and HF integral activity hotspots suggests e4 the best location for capturing sural and peroneal nerve-evoked SFs from both sides of the brainstem. The proximity of these two SF hotspots to e4 may explain why, collectively, these SFs performed with the highest-ranked feature-learnability at e4 compared to other electrode positions (compare feature-learnability in first two left columns between electrodes in Figure 5A). The positions of the remaining four hotspots in our earlier study correspond to a region rostral to e4, three of which were located on the left side between e4 and e1. This rostral and left-side bias of these activity hotspots residing outside e4’s surface territory may explain why feature-learnability was significantly greater when SFs were derived from left (compared to right) or rostral (compared to caudal) electrodes in the present study, and furthermore may explain why e1 was ranked next-best feature-learnability performer after e4. In further support for the idea that ML accuracy depends on electrode proximity to the current source, left (e1 and e2) and right (e6 and e7) electrodes generated smaller feature-learnability errors when predicting nerves from their ipsilateral side (see top row of Figure 3A). Together, these observations support the idea that regions of greater feature-learnability coincide with the location of signal generation within the DCN, which is likely to arise from achieving better signal quality when recording closer to the current source (Buzsáki et al., 2012).

### Determining the Most Efficient Electrode and Signal Feature Combination

We sought to determine which SFs and electrodes contributed the most information content to the ANNs, by achieving the highest decoding power from the minimum number of SFs and electrodes. If sensory information can be predicted from just a few SFs and electrodes, it not only reveals the most important, non-redundant, SFs and DCN recording locations, but it could also aid efficient targeting of DCN stimulation regions for evoking sensory percepts in the brain via a neuroprosthetic device.

Combinations of SFs and electrodes need to be carefully considered to avoid using inputs that provide redundant information or noise to the ANN. For example, with all 5 SFs included as inputs, 3 electrodes were the minimum required to achieve near-benchmark feature-learnability. Interestingly, we could reduce this to 2 electrodes (e{1, 4}) by excluding some SFs. Indeed, when each SF acquired from e4 was added to HF integral/e4 (Figure 4E), we found that the poorest ranked pair was HF integral paired with HF peak count, suggesting that the two HF SFs provide some overlapping or redundant information to the ANN. Consistent with this explanation was that correlation analysis revealed that HF integral and HF peak count were the highest correlated inputs. An example of the opposite case was also evident when HF SFs were added to P1N1 amplitude acquired from e{1–7}; the combination of P1N1 amplitude/e{1–7} (a LF SF) and HF integral/e{1–7} (a HF SF) achieved feature-learnability within 0.1% of the benchmark and outperformed combinations that included two LF SFs (i.e., P1N1 amplitude with N1 latency or P1N1 slope). Finally, by ranking feature-learnability of all possible combinations of SF pairs from e4 (Figure 5C), we demonstrated that combining one LF and one HF SF delivered significantly greater feature-learnability than SF pairs of the same frequency bands. These examples further suggest that the optimal feature-learnability combination requires at least one HF SF and one LF SF, which is similar to observations by Wong et al. (2016) when decoding cortical responses to retinal stimulation. This raises the question: what aspects of DCN neurophysiology do these HF and LF SFs represent, and why might they be complementary?

The LF components of local field potentials (LFPs) and their related electrocorticographic (ECoG) potentials (Buzsáki et al., 2012), similar to those investigated here, have been attributed to arise from slow network activity related to synaptic events (Buzsáki et al., 2012), voltage-dependent membrane oscillations (Kamondi et al., 1998), spike after potentials (Granit et al., 1963; Gustafsson, 1984), and Ca^2+^-mediated spikes (Wong et al., 1979; Logothetis, 2003). Combining electro-encephalographic (EEG) and intracortical recordings has revealed that the magnitude of slow potentials is related to the shape and size of dendritic arborizations near the recording electrodes, but not cell-size, as is the case for unit activity (Fromm and Bond, 1964, 1967; Logothetis, 2003). The HF components of neural ensemble recordings, obtained from a high-pass filter of ∼300–400 Hz (Logothetis, 2003), are believed to represent multiunit spiking activity generated from axonal action potentials. The magnitudes of HF and LF components are both affected by the proximity of the recording electrode. It has been suggested that summated spiking activity and individual spikes could be recorded up to 350 μm (Grover and Buchwald, 1970) and within ∼140 μm (Henze et al., 2000) from the recording electrode, respectively, but LFPs can be recorded up to 3 mm from a neural population (Mitzdorf, 1987; Logothetis, 2003). Consideration of both recording electrode location and the type of neurophysiological information represented by HF and LF potential components suggests that the ideal SF/electrode combination would acquire HF information from electrodes closest to axonal elements, and LF information from electrodes closest to dendro-somatic (nuclei) regions of neural ensembles. Future studies attempting to design DCN stimulation protocols to elicit meaningful sensory percepts to the cortex may need to carefully consider the choice of LF and HF stimulus features and the DCN location of the different stimuli presented. A future BMI would need to stimulate rather than record from the DCN, but currently, it is unclear how populations of neurons in the brainstem will be activated by different electrical stimulation protocols. For applications following spinal cord injury, a sensory BMI could read an artificial peripheral sensor, or decode neural signals from peripheral afferents or the spinal cord. These signals would then need to be transformed into a code used to stimulate the DCN, mimicking its activity as if it were receiving interpretable information from an intact spinal cord, and thereby bypassing a spinal lesion. Our study investigates the potential for DCN activity to be used for such a neural prosthetic device, i.e., we demonstrate that consistent, reliable signals are represented on the DCN surface. Our findings are therefore compatible with the idea that the DCN can be stimulated systematically to emulate the perception of sensory experiences arising from the periphery.

### DCN Feature-Learnability Asymmetry

A key finding from the present study was that information acquired from middle electrodes, particularly e4 and e5, could be used to predict nerve sides (left vs. right) with high accuracy. Each SF individually performed well above chance level (50%) for correctly predicting side when acquired from e4 (Figure 4D). This is striking because information from peripheral nerves is thought to remain ipsilateral at this level of the brainstem and activity is expected to be symmetrically expressed when evoked from the same nerve type on either side of the body. Our data indicates that information contained within the SFs obtained from the midline, under identical recording conditions (i.e., the same electrode), is significantly different when generated from opposite sides of the body. We provide evidence supporting this by demonstrating that side dominance (i.e., SF magnitude bias) acquired from e4 occurs in most (84%) cases across all SFs, animals and bilateral nerve pairs (Supplementary Figure S1B). This side dominance provides the necessary discrimination cues enabling the ML algorithm to correctly classify side from e4 SFs. This is supported by the SF/animal combinations that display side dominance in both sural and peroneal bilateral nerve pairs, which produce significantly fewer ML errors compared to those lacking a side dominance in at least one nerve pair (compare animals A2 and A5 of Supplementary Figure S1B). However, this concept is not specific to central electrodes. Discrimination enabling cues are present in the form of statistical differences between nerve SF magnitudes at different electrodes. This is demonstrated by the strong correlations between poorer feature-learnability outcomes and the total number of non-significant loci found between nerves for a given SF or electrode (Figure 6). Thus, the combinations of SF magnitudes across the different nerves at each electrode (Supplementary Figure S2) provides unique input/output patterns enabling feature-learnability to perform well above chance levels for side predictions, which furthermore, explains why adding inputs from additional electrodes generally improves feature-learnability accuracy as observed here and also by others (Mehring et al., 2003;Bansal et al., 2011; Wong et al., 2016).

What could cause the magnitude differences observed between bilateral nerve pairs recorded from e4? As all nerves were stimulated supramaximally, all nerve fibers were recruited and therefore side dominance is unlikely to result from an artifact of inadequate nerve stimulation on one side. Most animals expressed right-side dominance at the central electrode (e4) across all SFs (note left-side N1 latency dominance indicates shorter right-side latencies), including those with the most left- and right-shifted electrodes (Supplementary Figure S1B). It is therefore unlikely that electrode misalignment over the midline contributed to signal side dominance. Our favored explanation for the SF magnitude asymmetry is that these signals are asymmetrically represented across the brainstem surface. We previously reported that there were no significant differences between two SFs (same SFs used in the present study) when generated from bilateral nerve pairs and recorded from their hotspot locations (Loutit et al., 2017). However, the hotspot locations were asymmetrically aligned across the DCN. Considering these observations, we propose that the high accuracy of ML derived from midline electrodes result from a combination of (i) the electrode being near the current source, which as discussed above, provides better signal quality, and (ii) the asymmetric distribution of current sources generated by bilateral nerve pairs provide magnitude difference cues between the two sides. Recent evidence suggests that asymmetries in gene expression in the spinal cord may lead to hemispheric asymmetries (Ocklenburg et al., 2017). It follows that nuclei along the motor and sensory pathways between the spinal cord and cortex might also be organized asymmetrically. The underlying genetic and epigenetic factors involved, to our knowledge, have not been characterized in rats. Future studies investigating the possibility of lateralization of neural structures within the DCN are therefore warranted.

## Conclusion

Stimulating the periphery evokes unique electrical signatures across the DCN surface that can be decoded using ML. The ability to train an ANN on input features obtained from one set of animals and achieve high classification accuracy based on data from other animals, demonstrates that electrical nerve stimulation produces similar patterns of neural activity in the DCN across different animals. Furthermore, we show that feature-learnability is a powerful approach for identifying information-rich locations and useful SFs, as well as determining the minimum information required for accurate decoding of sensory signals. Our capacity to identify information-rich SFs generated from unique stimulation patterns may provide new insights and approaches for the future development of somatosensory neural prostheses. Our evidence that peripherally evoked DCN activity is asymmetrically represented across the brainstem surface challenges the current view of brainstem organization and suggests a lateralization of neural structures.

## Data Availability

The datasets generated for this study are available on request to the corresponding author.

## Author Contributions

JP conceived and designed the study. AL conducted the research. AL, JP, and TM contributed analytical tools. AL and JP analyzed the data. AL, JP, MS, TM, SR, GS, JM, IB, and RV wrote the manuscript.

## Conflict of Interest Statement

The authors declare that the research was conducted in the absence of any commercial or financial relationships that could be construed as a potential conflict of interest.

## Abbreviations

ANN: artificial neural network
DCN: dorsal column nuclei
e1: electrode 1
e2: electrode 2
e3: electrode 3
e4: electrode 4
e5: electrode 5
e6: electrode 6
e7: electrode 7
e{1–7}: inclusive of electrodes 1 to 7
FDR: false discovery rate
HF: high-frequency
LF: low-frequency
LM: linear regression model
LMEM: linear mixed-effects model
LOO: leave one out
LPN: left peroneal nerve
LSN: left sural nerve
ML: machine learning
N1: peak of the first major negative deflection of the dorsal column nuclei surface potential
P1: peak of the first major positive deflection of the dorsal column nuclei surface potential
P1N1: height from the peak of the first major positive deflection to the peak of the first major negative deflection of the dorsal column nuclei surface potential
PP: pooled population
RPN: right peroneal nerve
RSN: right sural nerve
S1: primary somatosensory cortex
SEM: standard error of the mean
SF: signal feature
sMEA: surface multi-electrode array
TRM: total response magnitude
WIA: within individual animals.
