# Supplementary material for: Peripheral Nerve Activation Evokes Machine-Learnable Signals in the Dorsal Column Nuclei
Source: Front Syst Neurosci. 2019 Mar 20;13:11. doi: 10.3389/fnsys.2019.00011 (PMC6448039; doi:10.3389/fnsys.2019.00011)
Supplement: Supplementary file 1 [file Data_Sheet_1.pdf]

Supplementary Material

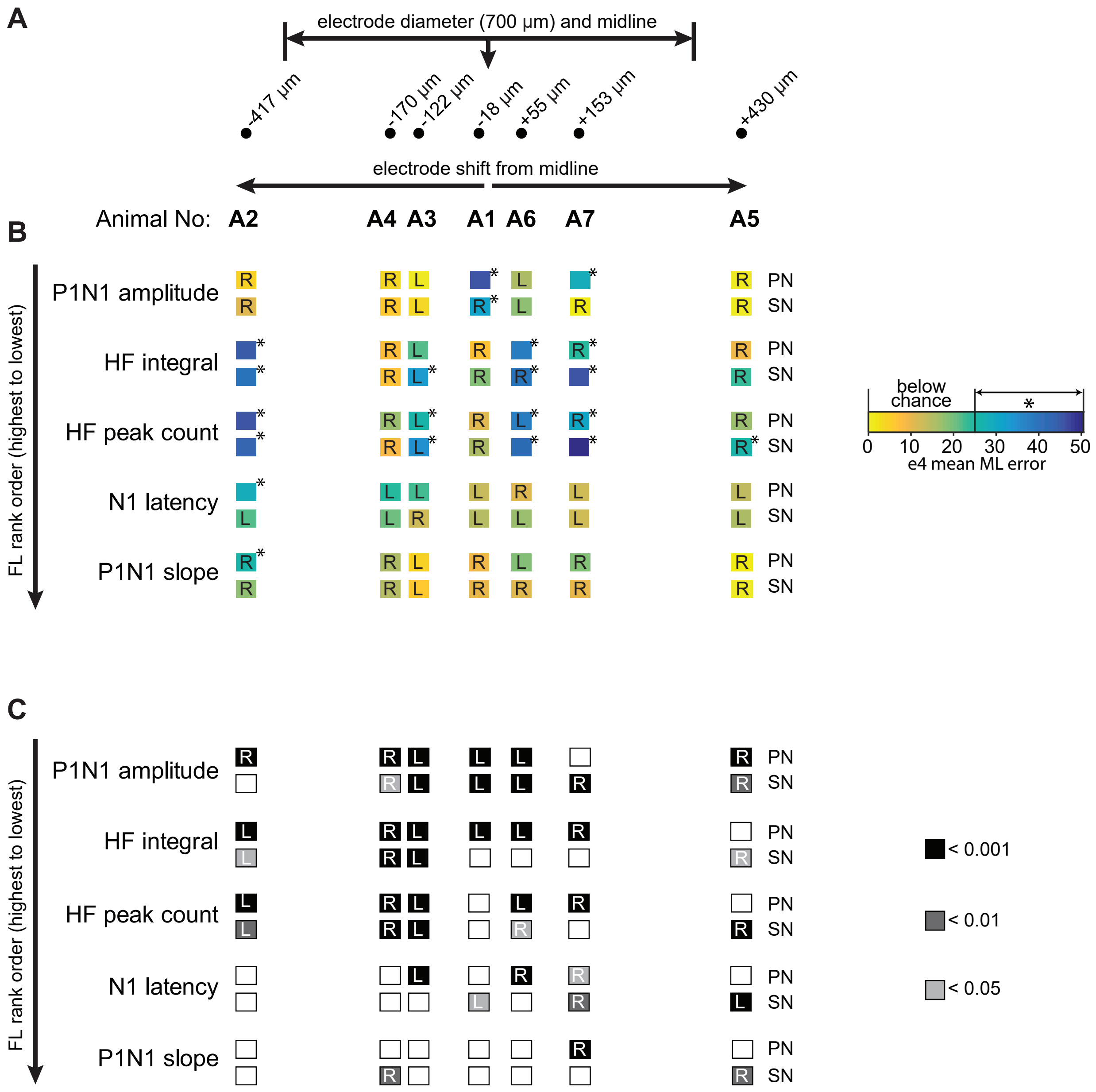

Figure S1. Electrode shift, and e4 and Total Response Magnitude side dominance. (A) Electrode shift: The top line represents the dimensions of midline electrodes (downward arrow indicating the midline) of an ideal placed sMEA. The dots below this line indicate the relative shift off the midline for each animal (dots represent electrode centre; numbers indicate the shift magnitudes and directions

relative to the midline; animals labelled as indicated A1-A5). Two approaches were used to establish if left and right nerves of bilateral nerve pairs evoked SFs of significantly different magnitudes, referred to as a *side dominance*. Signal side dominance results (**B** and **C**) for each animal are aligned in columns according to electrode shift. **(B) e4 side dominance:** For each bilateral nerve pair (i.e. peroneal or sural nerve), an L or R was designated to the animal when a SF's magnitude, recorded from e4, was significantly greater when evoked on the left or right side, respectively. Each SF, ordered in rank from greatest to least feature-learnability, is split into two rows; one for each bilateral nerve pair (indicated at end of each row; sural nerve, SN; peroneal nerve, PN). The coloured squares indicate the mean machine learning (ML) error (see Materials and Methods) of the bilateral nerve pairs, where cooler colours indicate greater ML errors. Asterisks indicate for each bilateral nerve pair where ML errors were, on average, greater than chance level (25%, i.e. cooler colours). Note that i) most animals (except A6) were right dominant regardless of electrode shift (larger N1 latency indicates shorter right-side latencies); ii) occurrences of larger e4 mean ML errors (asterisks) are more often accompanied by a lack of e4 side dominance in one or both bilateral nerve pairs. **(C) Total Response Magnitudes:** In another approach, the dominant side of a SF was determined using the total response magnitude (TRM). To calculate TRMs, SF values from each of the 7 electrodes of individual trials were summed together, and the means of these sums were calculated from 200-220 trials for each SF and nerve. Thus, the TRM for each SF/animal is the mean of all respective sums (i.e. all the values at e1-e7 for each animal and SF) that is evoked by a single nerve. L and R indicate the side for each bilateral nerve pair (and animal and SF) for which the TRM was significantly greater (significant levels indicated by shading) than its bilateral nerve counterpart. Note that the rows of higher ranked SFs have greater numbers of significant side-dominant TRMs (i.e. more shaded boxes).

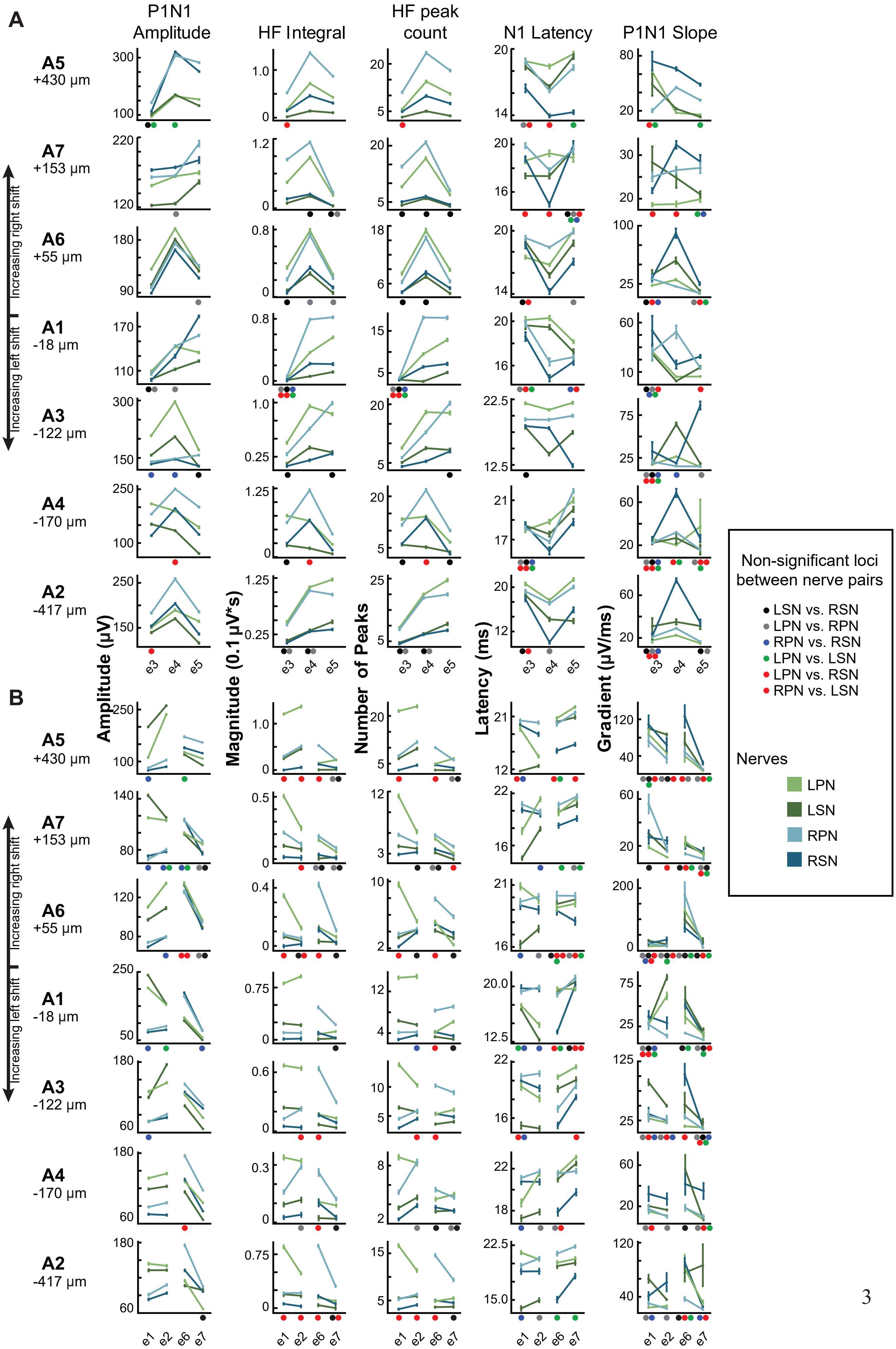

A
B
P1N1 Amplitude
HF Integral
HF peak count
N1 Latency
P1N1 Slope
A5 +430 µm
A7 +153 µm
A6 +55 µm
A1 -18 µm
A3 -122 µm
A4 -170 µm
A2 -417 µm
Increasing right shift
Increasing left shift
Amplitude (µV)
Magnitude (0.1 µV*s)
Number of Peaks
Latency (ms)
Gradient (µV/ms)
e3 e4 e5
e1 e2 e6 e7
Non-significant loci between nerve pairs
LSN vs. RSN
LPN vs. RPN
RPN vs. RSN
LPN vs. LSN
LPN vs. RSN
RPN vs. LSN
Nerves
LPN
LSN
RPN
RSN

Figure S2. Signal feature quantification and determining non-significant loci as potential errors for ANN classification. (A) Line plots generated for each of the 7 animals (A1-A7) show the mean ± SEM magnitude of each SF (200-220 values per nerve), extracted from nerve-evoked DCN signals (one SF per column of graphs), recorded from the middle electrodes (e3, e4 and e5, x-axis). Each row of graphs represents data from one animal, which are ordered vertically by the magnitude of sMEA shift from the midline (as indicated to the left of the graphs, see also **Figure S1**). Line colours indicate different nerves (as per legend, insert). Coloured dots below each graph indicate classification of non-significant loci into one of 5 possible categories, i.e. two nerve types not significantly different (as per the figure key) at an electrode site. **(B)** Same as **(A)**, but SFs were extracted from left- (e1 and e2) and right-sided (e6 and e7) electrodes of the sMEA. Note i) the occurrences of more dots at lower ranked signal features, see **Figure 6** for quantification of these dots and correlations to feature-learnability; ii) electrode shift has no clear effect on responses; iii) functional symmetry would be observed by the occurrence of parallel lines in **(B)**, i.e. lines between e1 and e2 would be parallel to lines between e5 and e6. See **Figure 3** for abbreviations.
